# Supplementary figures and images for: Detectability of runs of homozygosity is influenced by analysis parameters and population-specific demographic history
Source: PLoS Comput Biol. 2024 Oct 31;20(10):e1012566. doi: 10.1371/journal.pcbi.1012566 (PMC11556709; doi:10.1371/journal.pcbi.1012566)

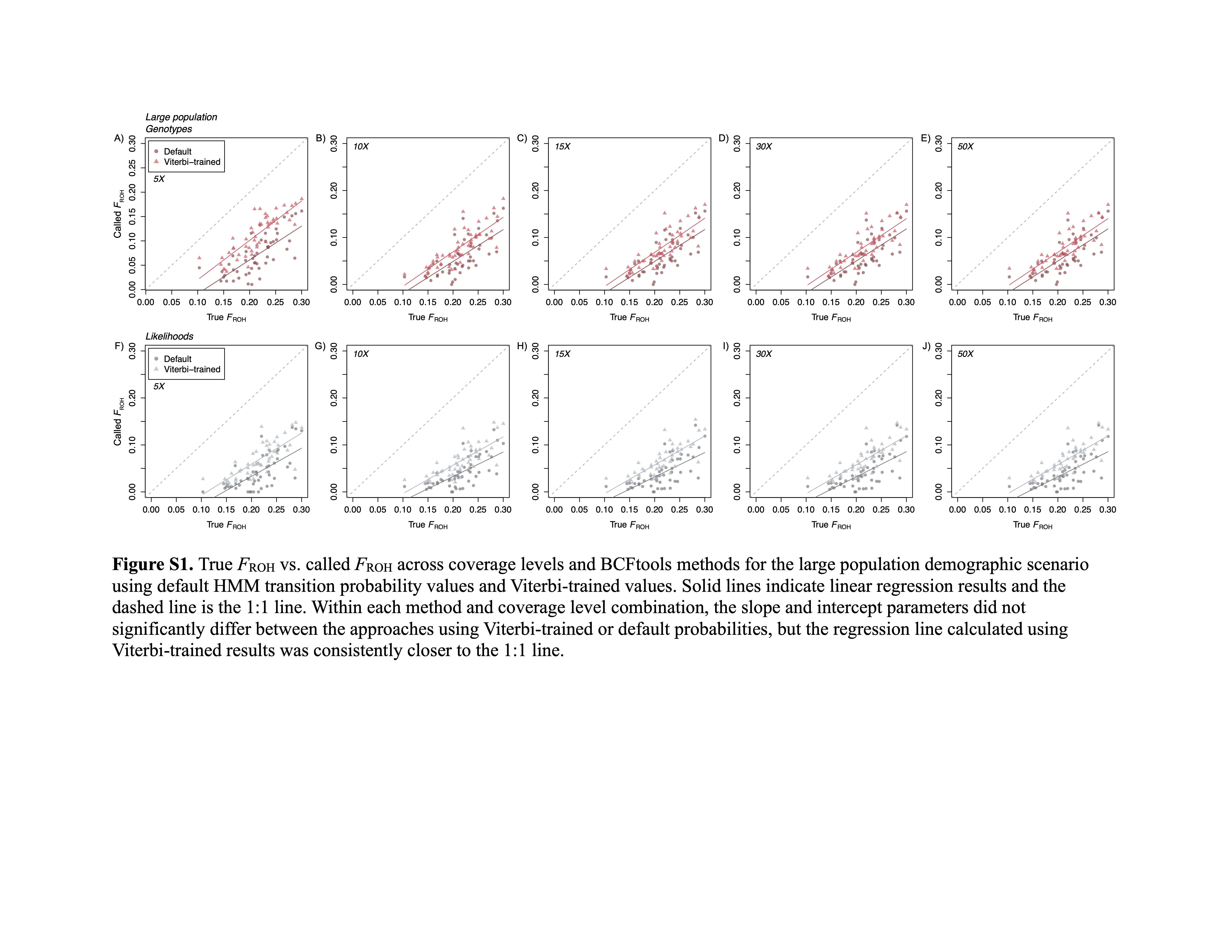

Supplement: S1 Fig — Solid lines indicate linear regression results and the dashed line is the 1:1 line. Within each method and coverage level combination, the slope and intercept parameters did not significantly differ between the approaches using Viterbi-trained or default probabilities, but the regression line calculated using Viterbi-trained results was consistently closer to the 1:1 line. (TIFF) [file pcbi.1012566.s007.tiff]

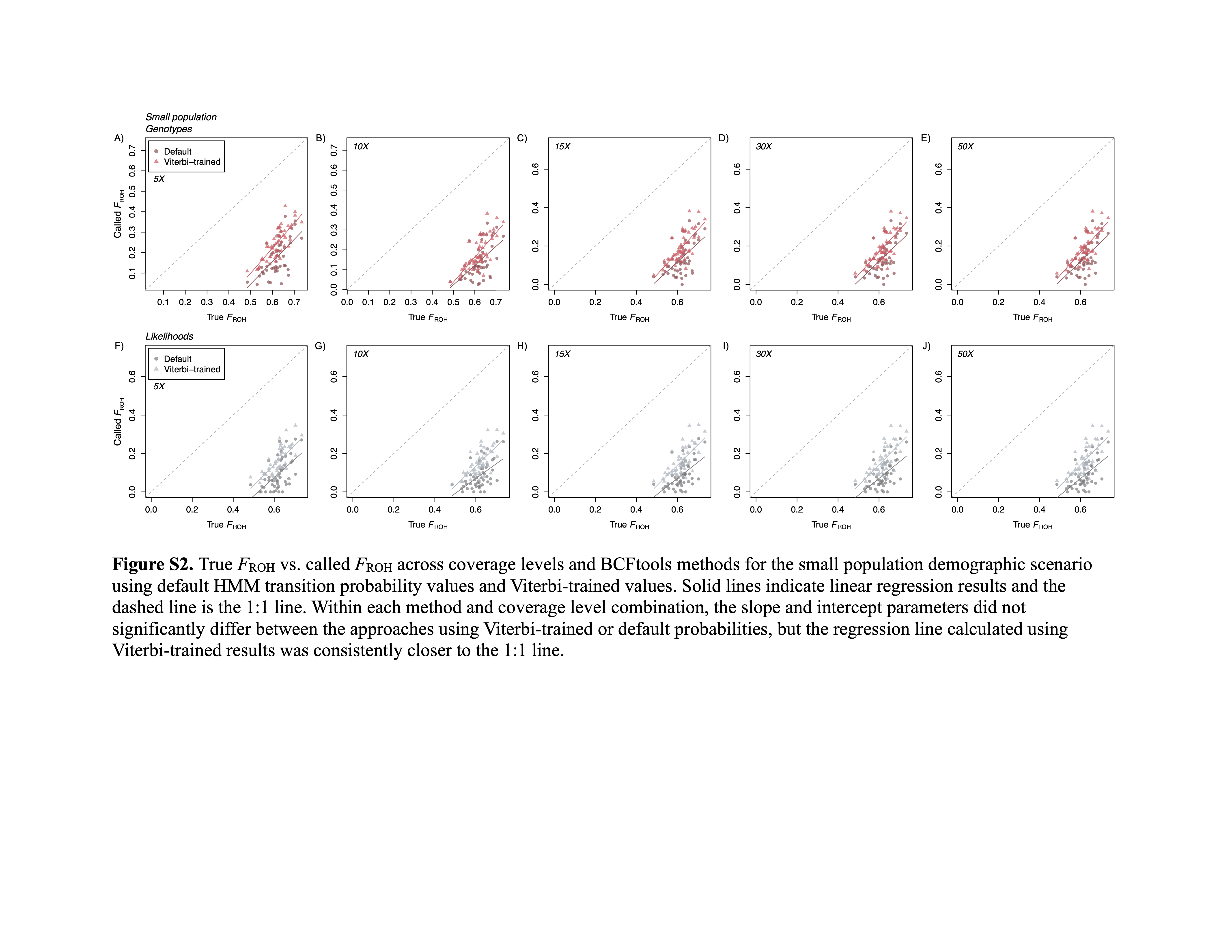

Supplement: S2 Fig — Solid lines indicate linear regression results and the dashed line is the 1:1 line. Within each method and coverage level combination, the slope and intercept parameters did not significantly differ between the approaches using Viterbi-trained or default probabilities, but the regression line calculated using Viterbi-trained results was consistently closer to the 1:1 line. (TIFF) [file pcbi.1012566.s008.tiff]

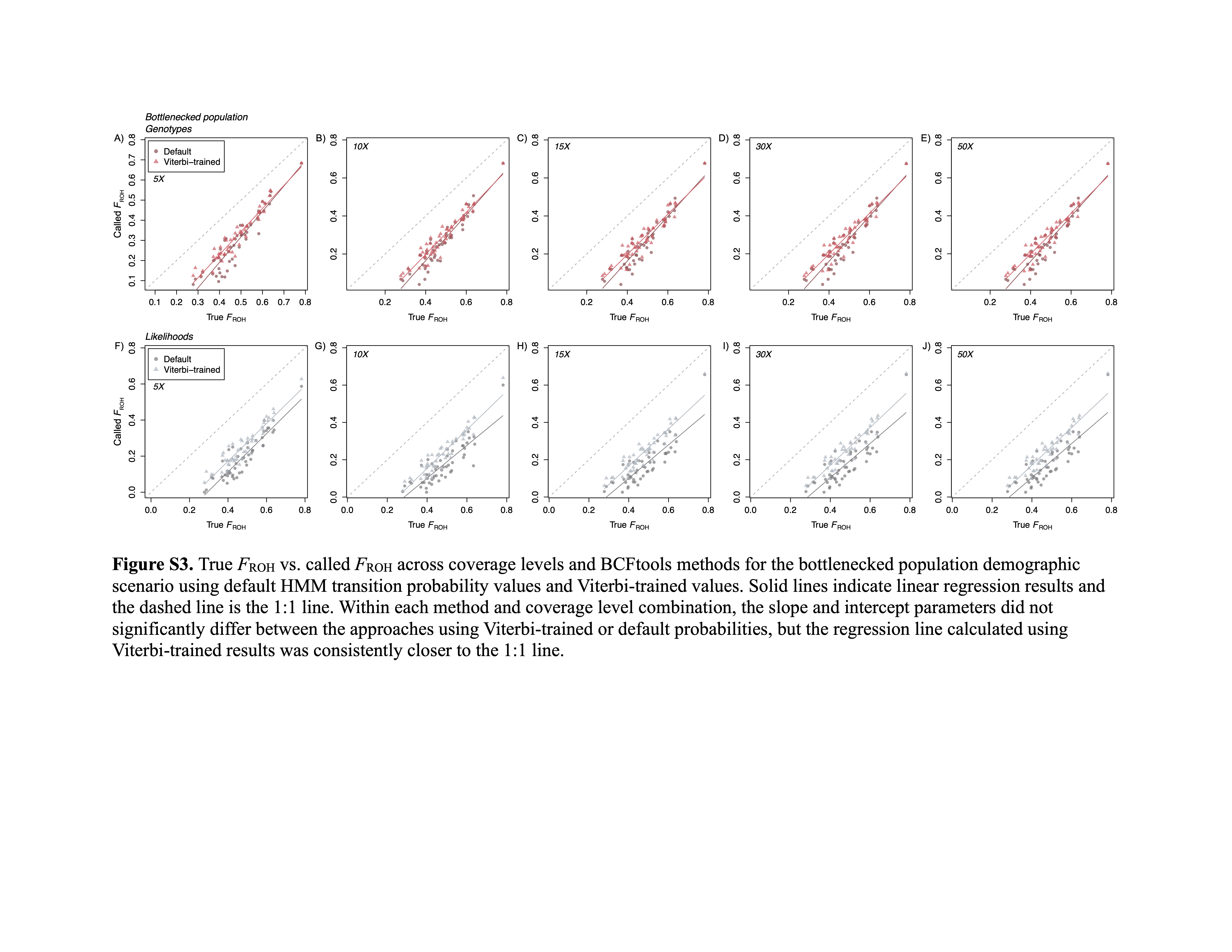

Supplement: S3 Fig — Solid lines indicate linear regression results and the dashed line is the 1:1 line. Within each method and coverage level combination, the slope and intercept parameters did not significantly differ between the approaches using Viterbi-trained or default probabilities, but the regression line calculated using Viterbi-trained results was consistently closer to the 1:1 line. (TIFF) [file pcbi.1012566.s009.tiff]

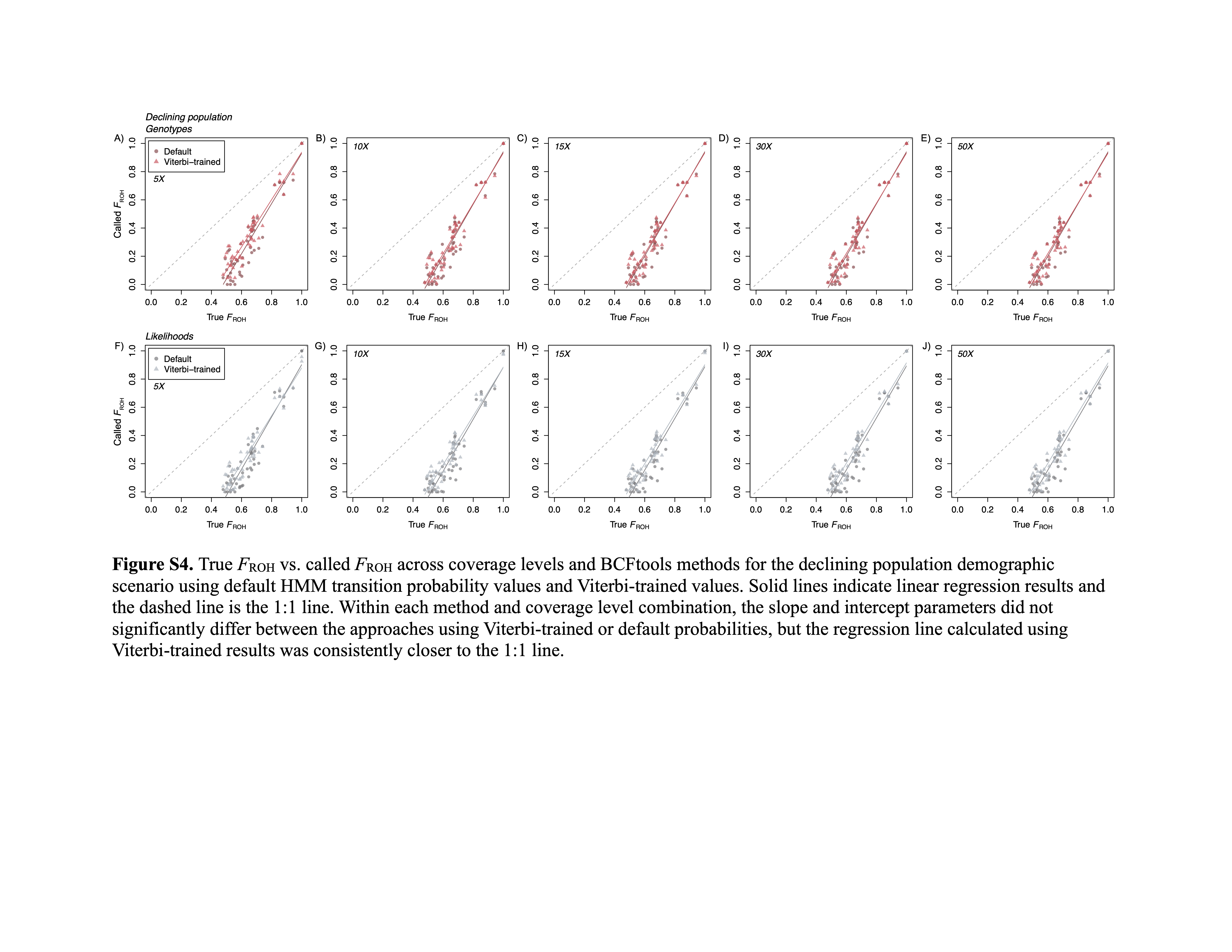

Supplement: S4 Fig — Solid lines indicate linear regression results and the dashed line is the 1:1 line. Within each method and coverage level combination, the slope and intercept parameters did not significantly differ between the approaches using Viterbi-trained or default probabilities, but the regression line calculated using Viterbi-trained results was consistently closer to the 1:1 line. (TIFF) [file pcbi.1012566.s010.tiff]

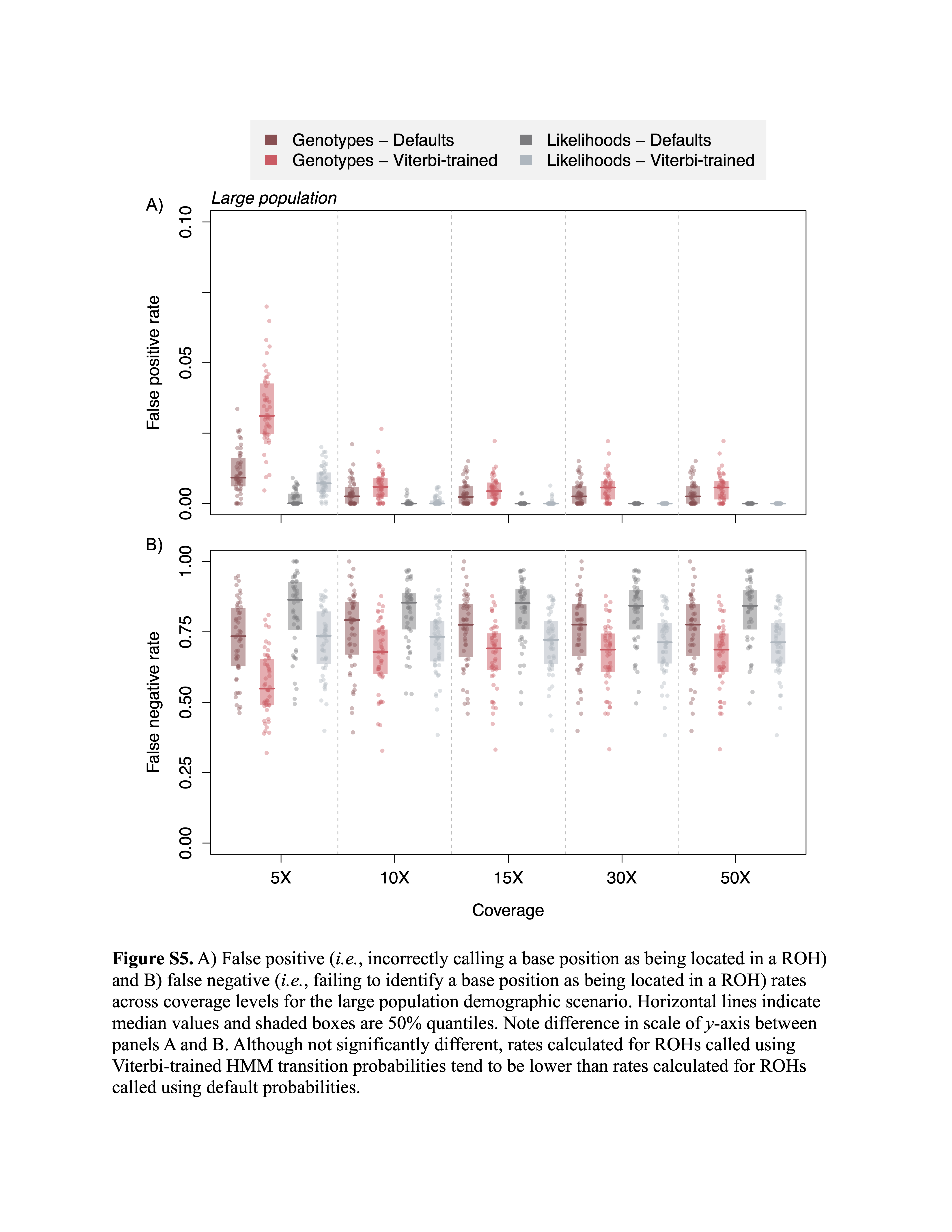

Supplement: S5 Fig — A) False positive (i.e., incorrectly calling a base position as being located in a ROH) and B) false negative (i.e., failing to identify a base position as being located in a ROH) rates across coverage levels for the large population demographic scenario. Horizontal lines indicate median values and shaded boxes are 50% quantiles. Note difference in scale of y-axis between panels A and B. Although not significantly different, rates calculated for ROHs called using Viterbi-trained HMM transition probabilities tend to be lower than rates calculated for ROHs called using default probabilities. (TIFF) [file pcbi.1012566.s011.tiff]

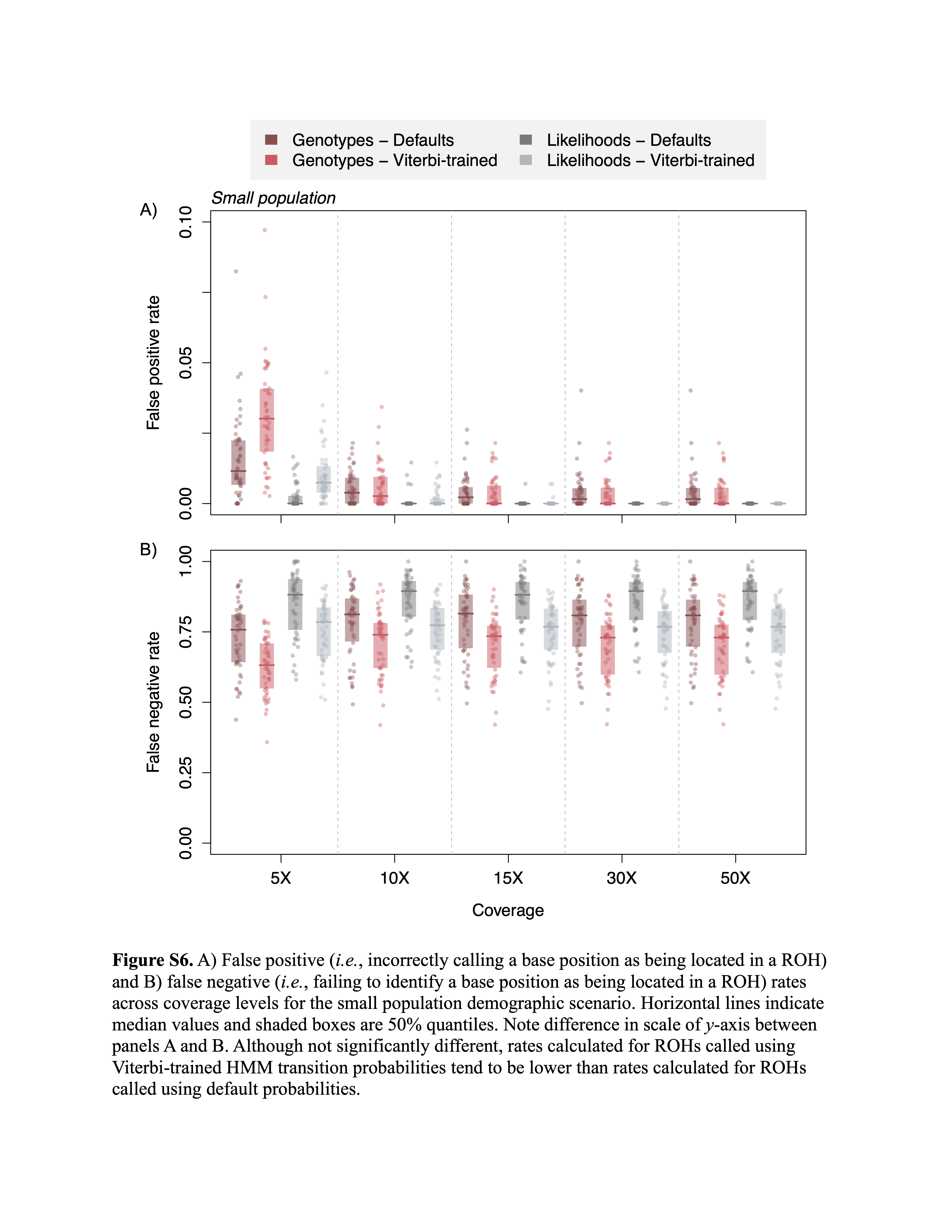

Supplement: S6 Fig — A) False positive (i.e., incorrectly calling a base position as being located in a ROH) and B) false negative (i.e., failing to identify a base position as being located in a ROH) rates across coverage levels for the small population demographic scenario. Horizontal lines indicate median values and shaded boxes are 50% quantiles. Note difference in scale of y-axis between panels A and B. Although not significantly different, rates calculated for ROHs called using Viterbi-trained HMM transition probabilities tend to be lower than rates calculated for ROHs called using default probabilities. (TIFF) [file pcbi.1012566.s012.tiff]

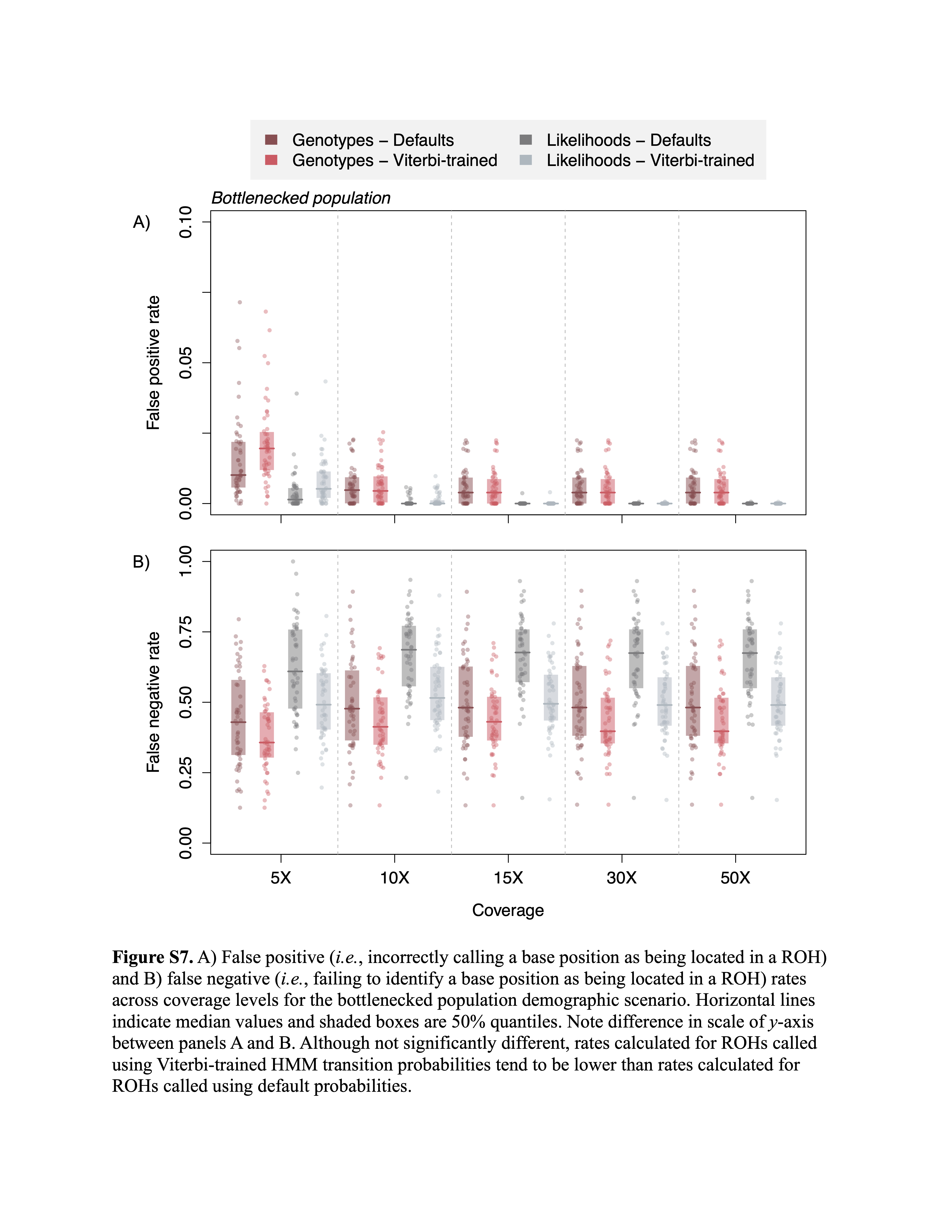

Supplement: S7 Fig — A) False positive (i.e., incorrectly calling a base position as being located in a ROH) and B) false negative (i.e., failing to identify a base position as being located in a ROH) rates across coverage levels for the bottlenecked population demographic scenario. Horizontal lines indicate median values and shaded boxes are 50% quantiles. Note difference in scale of y-axis between panels A and B. Although not significantly different, rates calculated for ROHs called using Viterbi-trained HMM transition probabilities tend to be lower than rates calculated for ROHs called using default probabilities. (TIFF) [file pcbi.1012566.s013.tiff]

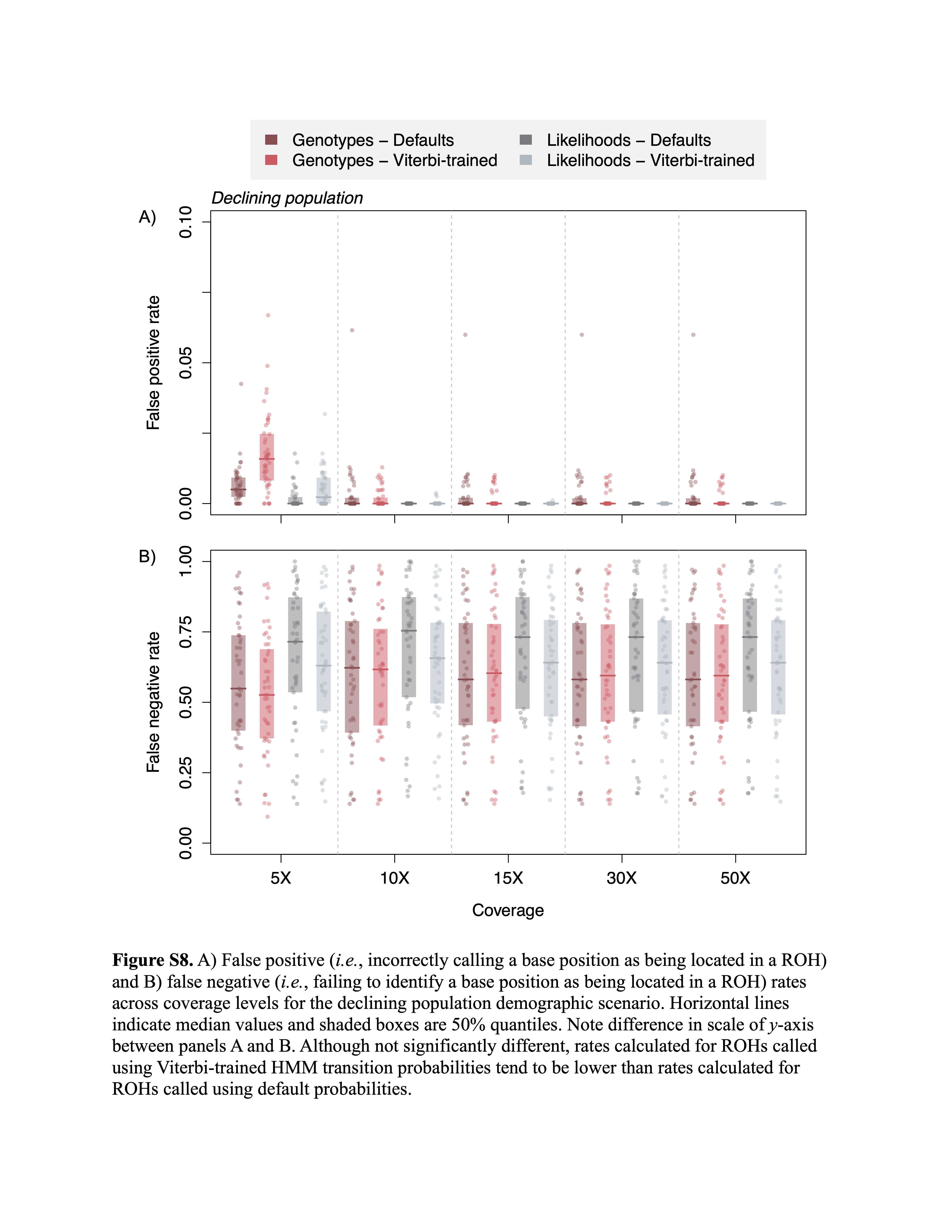

Supplement: S8 Fig — A) False positive (i.e., incorrectly calling a base position as being located in a ROH) and B) false negative (i.e., failing to identify a base position as being located in a ROH) rates across coverage levels for the declining population demographic scenario. Horizontal lines indicate median values and shaded boxes are 50% quantiles. Note difference in scale of y-axis between panels A and B. Although not significantly different, rates calculated for ROHs called using Viterbi-trained HMM transition probabilities tend to be lower than rates calculated for ROHs called using default probabilities. (TIFF) [file pcbi.1012566.s014.tiff]

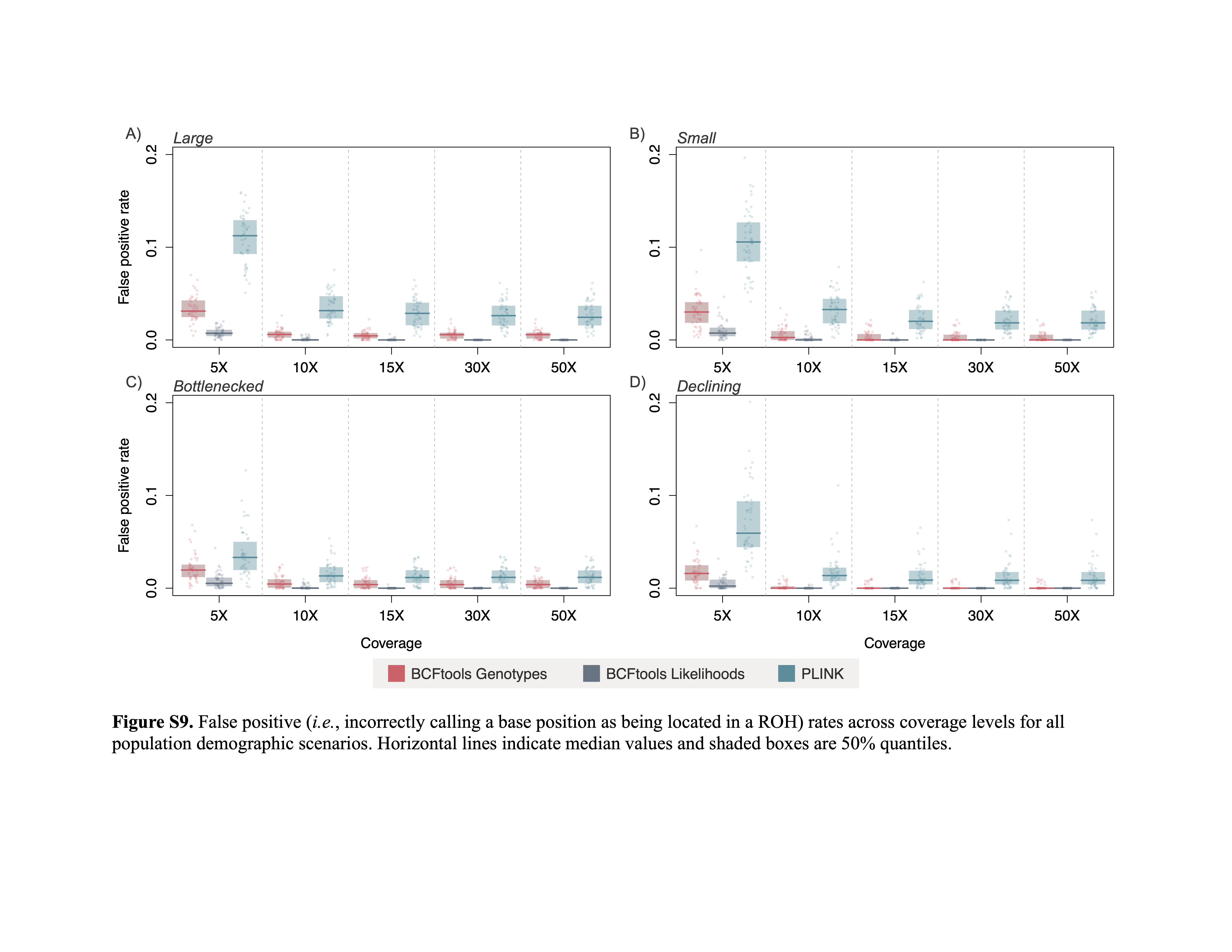

Supplement: S9 Fig — Horizontal lines indicate median values and shaded boxes are 50% quantiles. (TIFF) [file pcbi.1012566.s015.tiff]

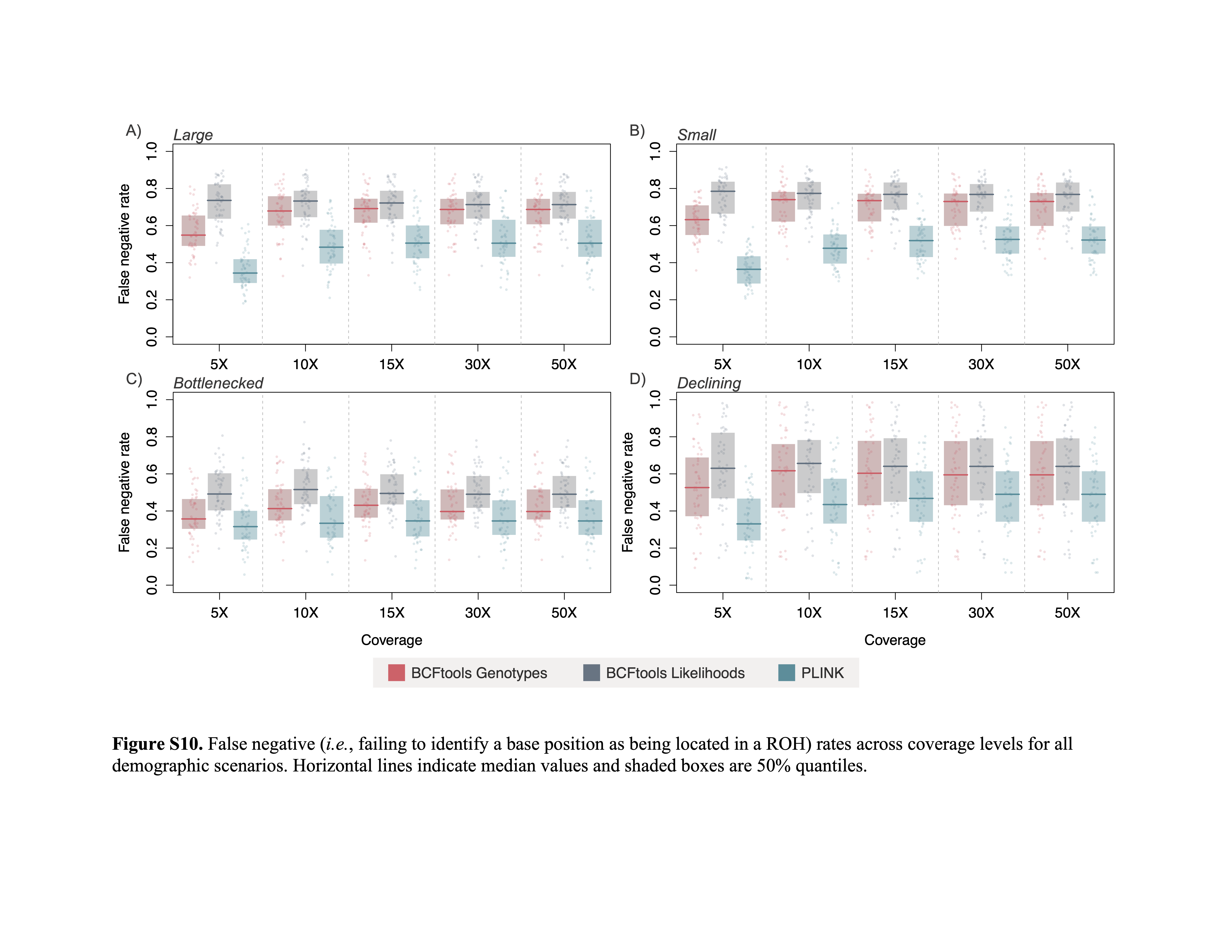

Supplement: S10 Fig — Horizontal lines indicate median values and shaded boxes are 50% quantiles. (TIFF) [file pcbi.1012566.s016.tiff]

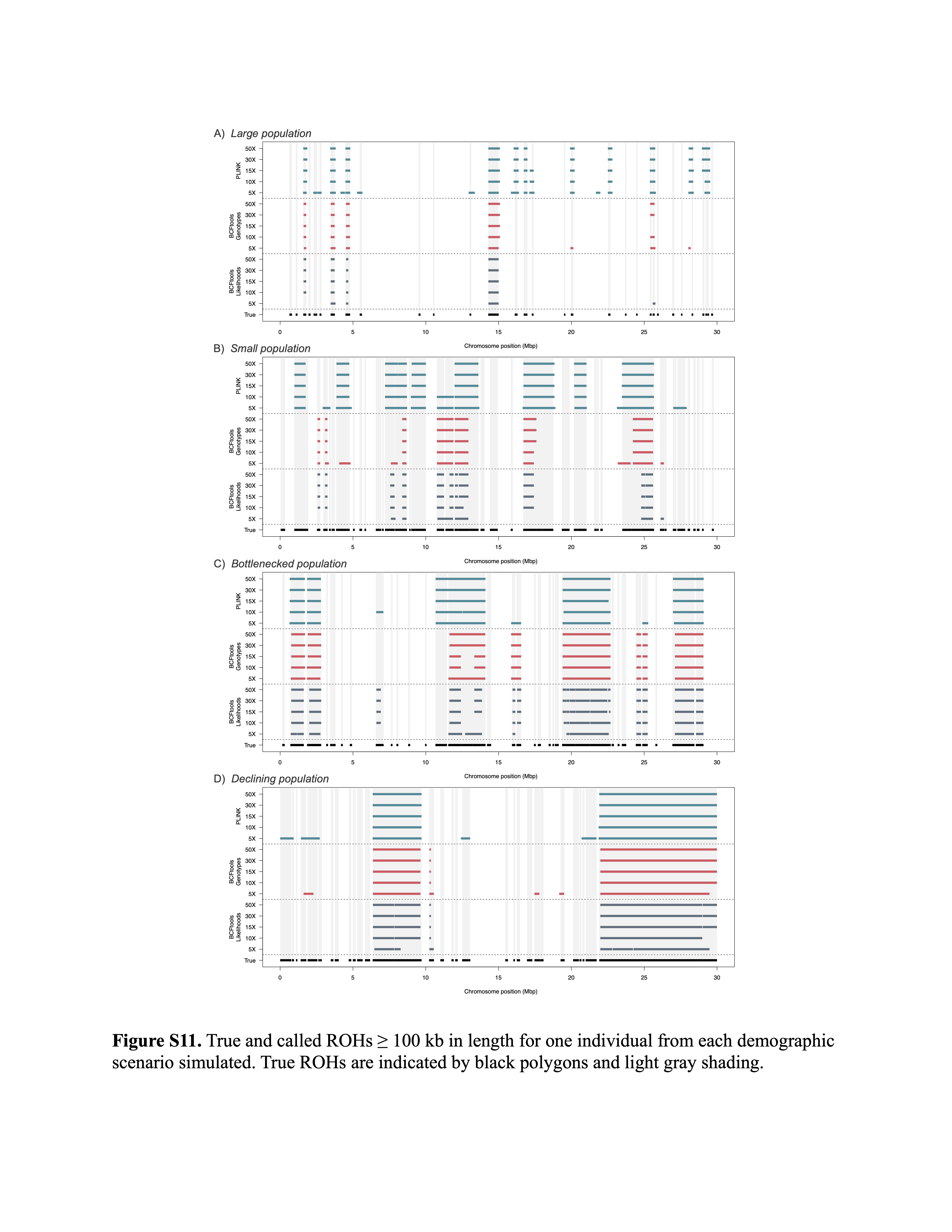

Supplement: S11 Fig — True ROHs are indicated by black polygons and light gray shading. (TIFF) [file pcbi.1012566.s017.tiff]

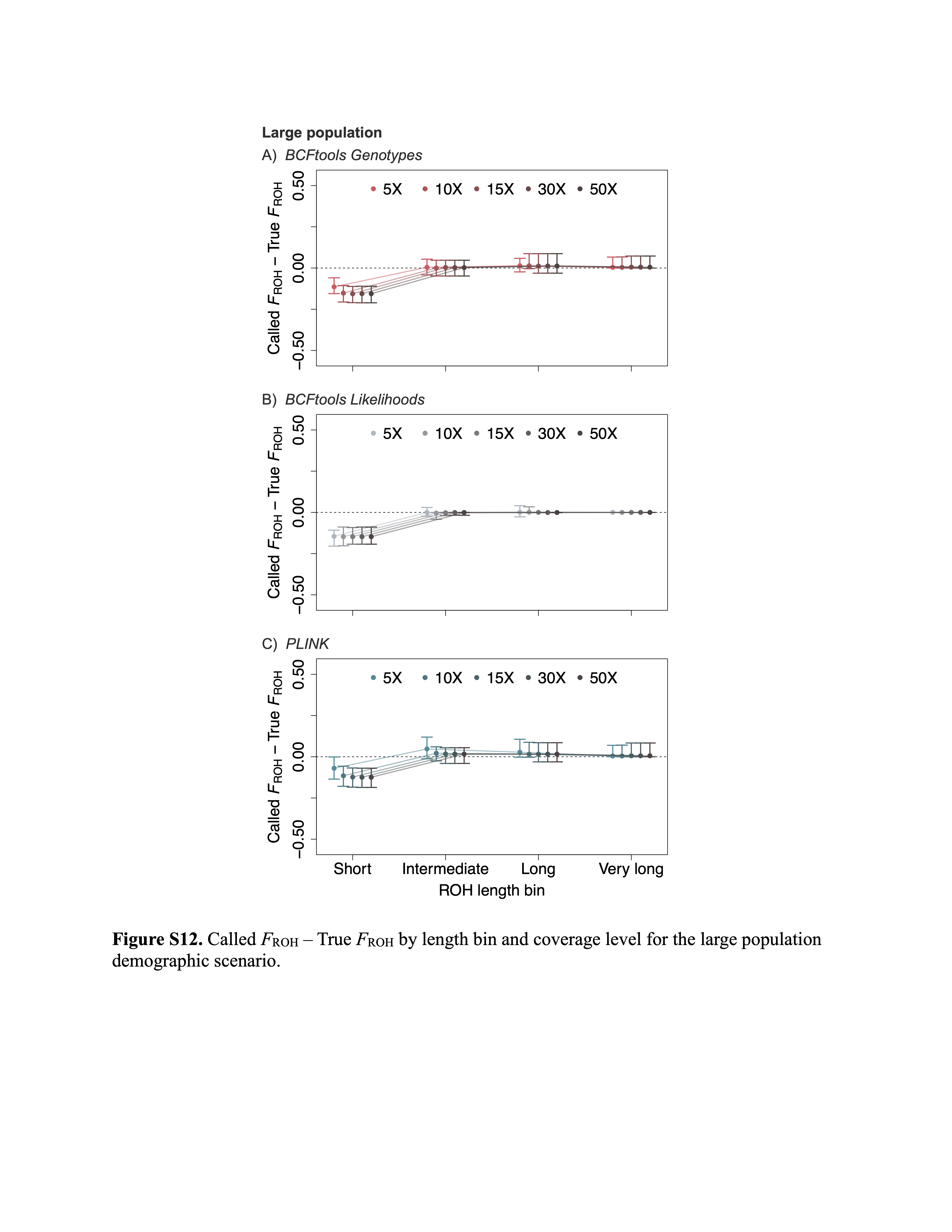

Supplement: S12 Fig — (TIFF) [file pcbi.1012566.s018.tiff]

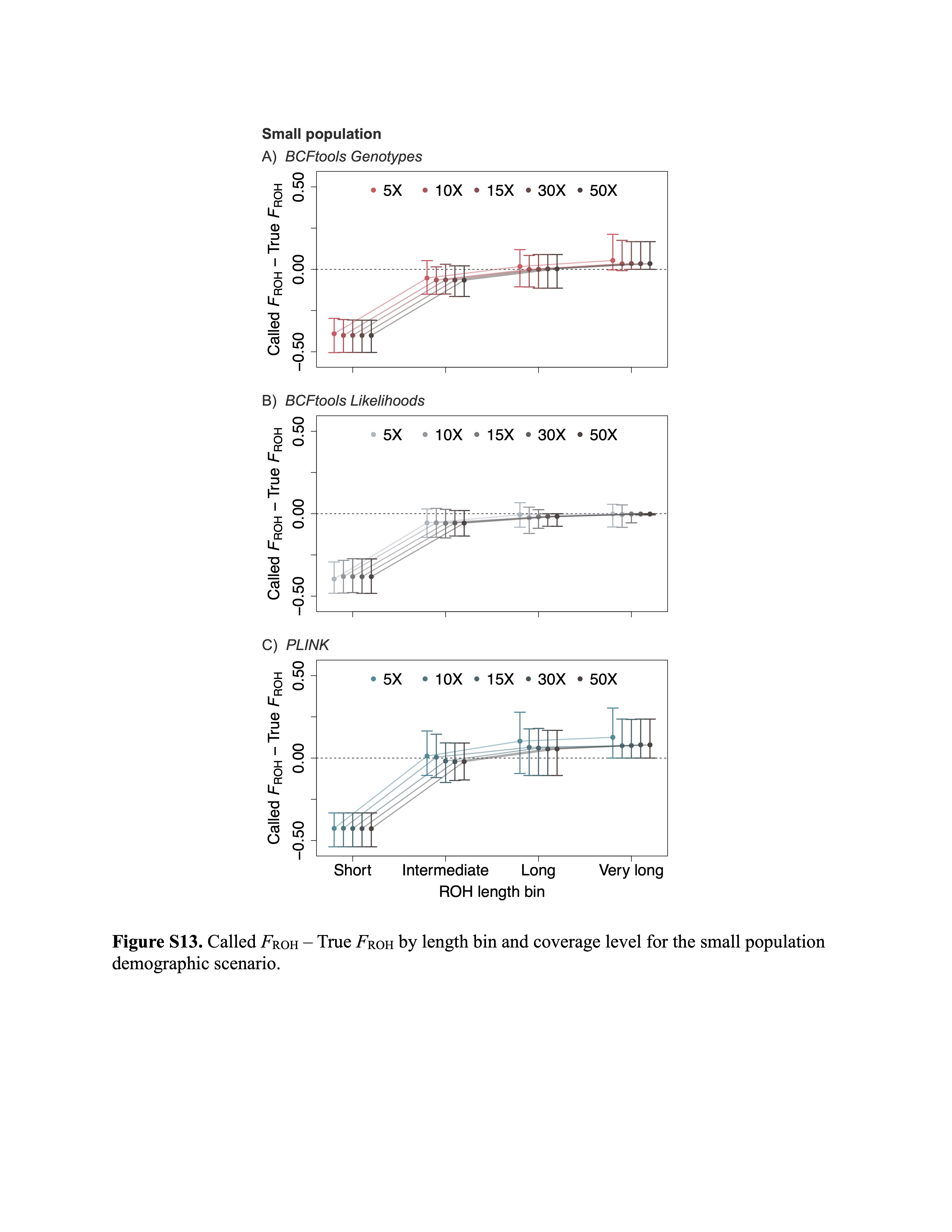

Supplement: S13 Fig — (TIFF) [file pcbi.1012566.s019.tiff]

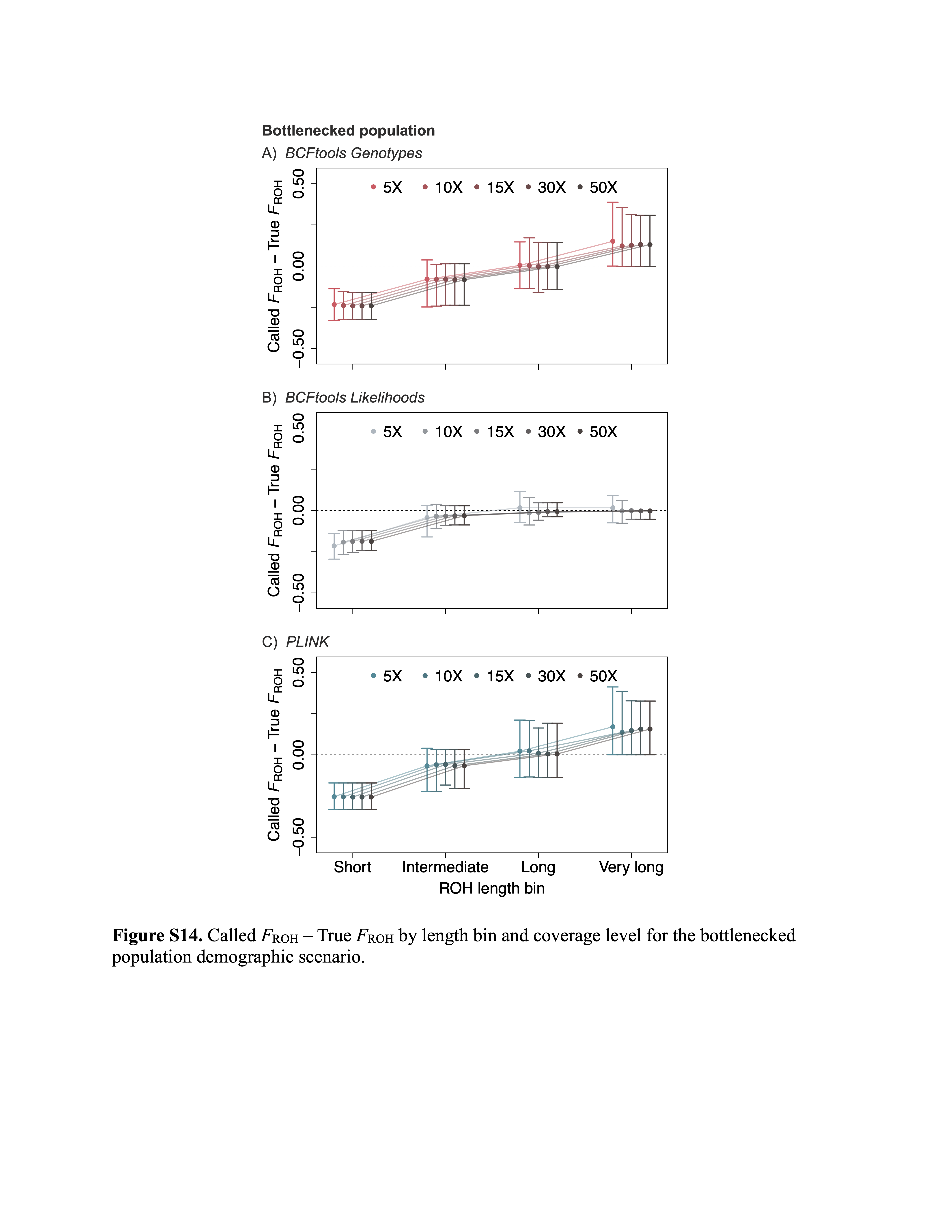

Supplement: S14 Fig — (TIFF) [file pcbi.1012566.s020.tiff]

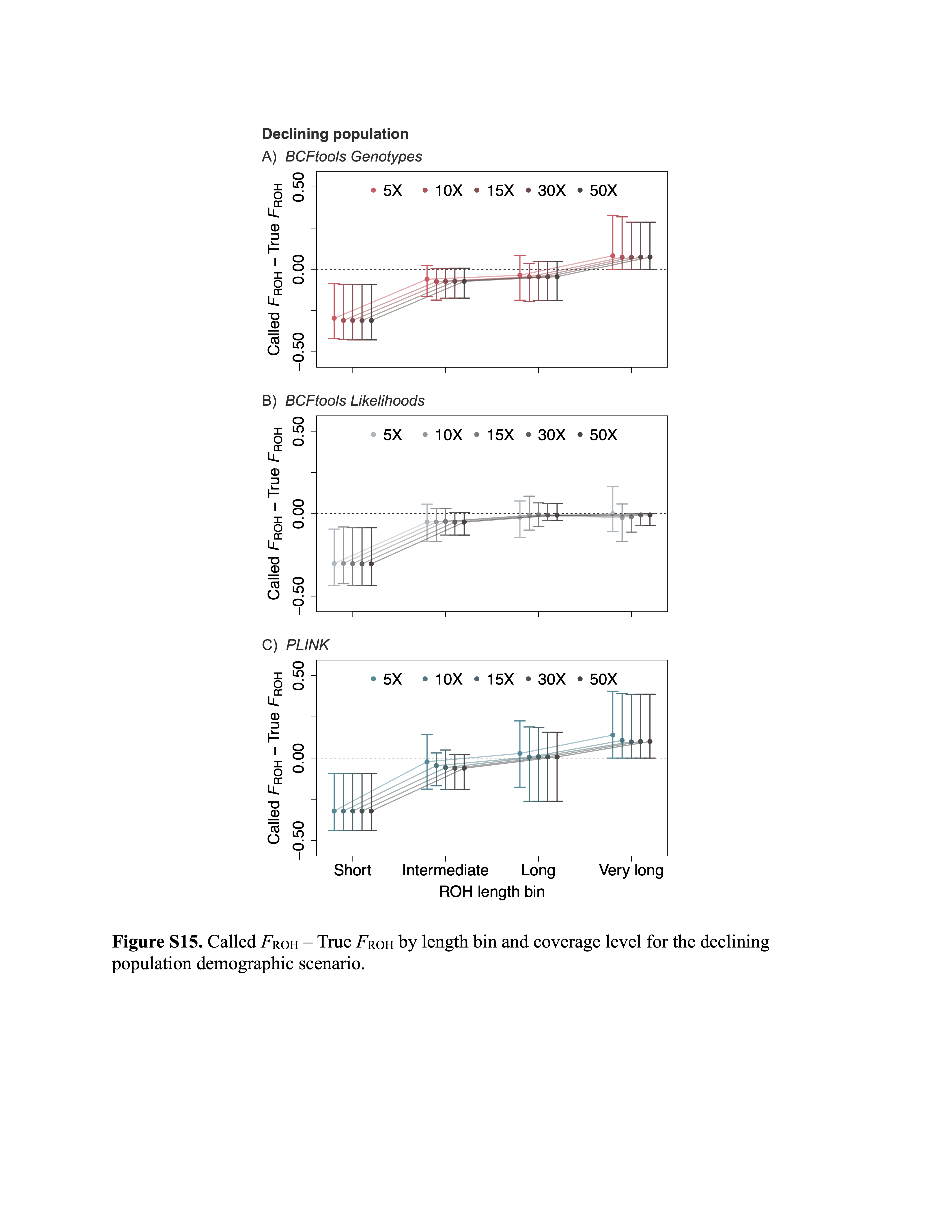

Supplement: S15 Fig — (TIFF) [file pcbi.1012566.s021.tiff]

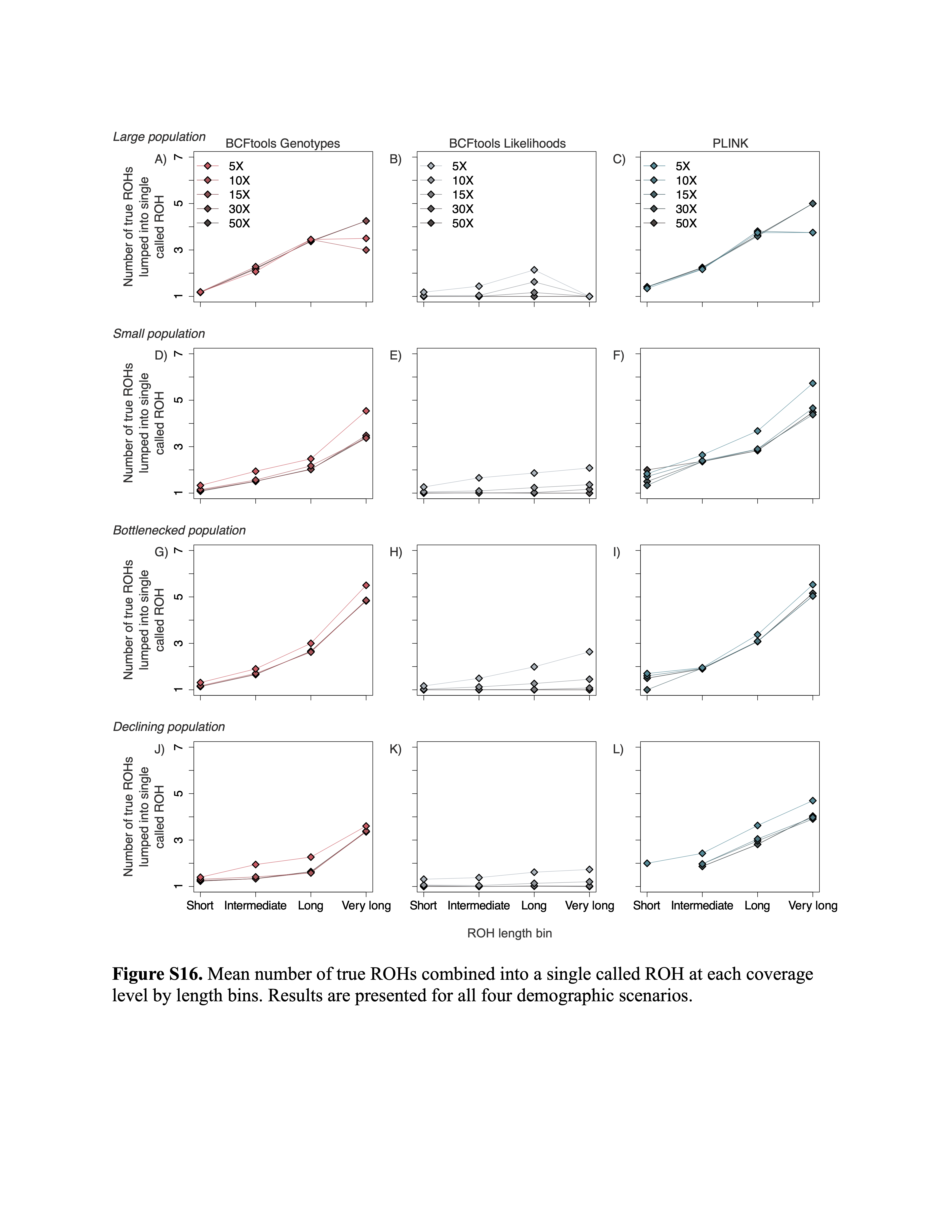

Supplement: S16 Fig — Results are presented for all four demographic scenarios. (TIFF) [file pcbi.1012566.s022.tiff]

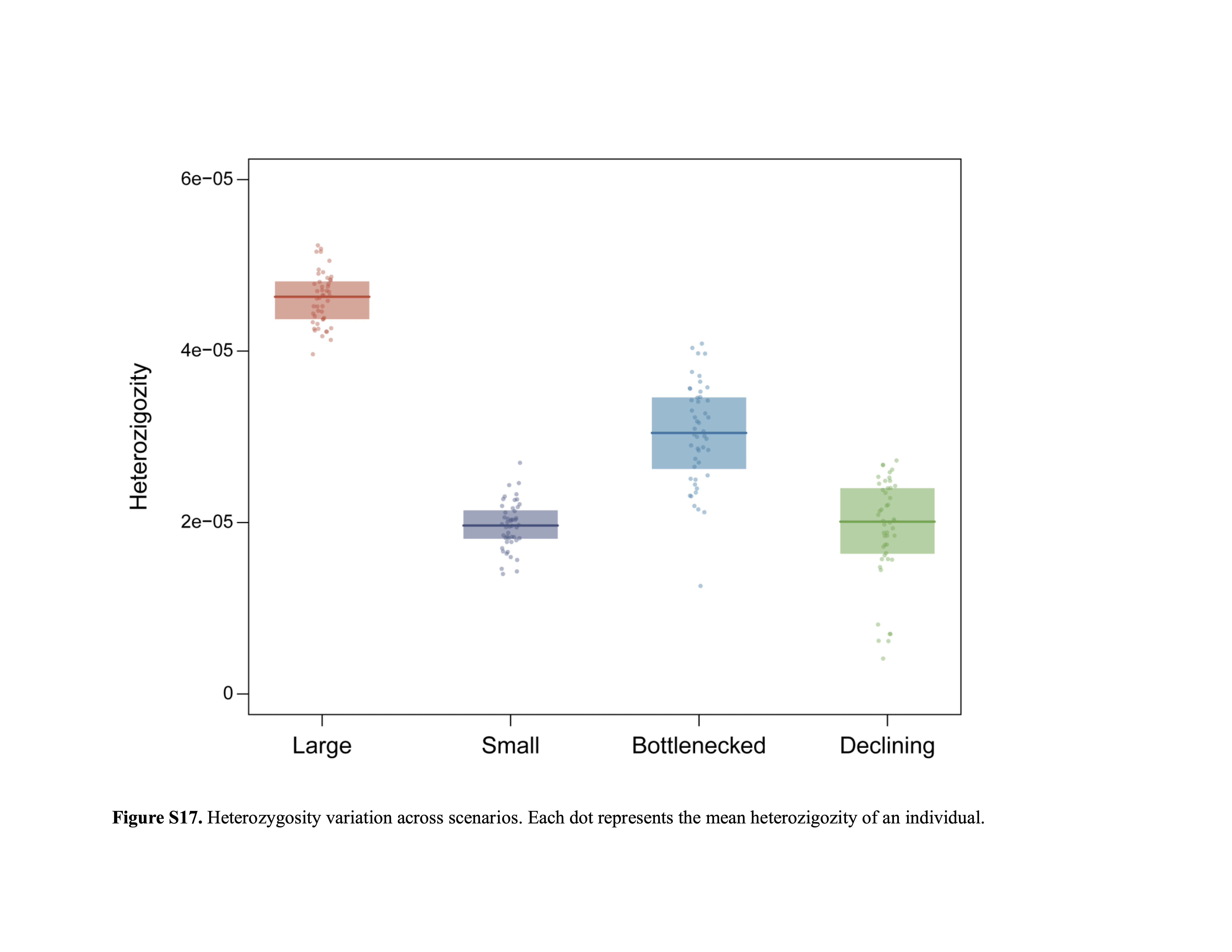

Supplement: S17 Fig — Each dot represents the mean heterozygosity of an individual. (TIFF) [file pcbi.1012566.s023.tiff]

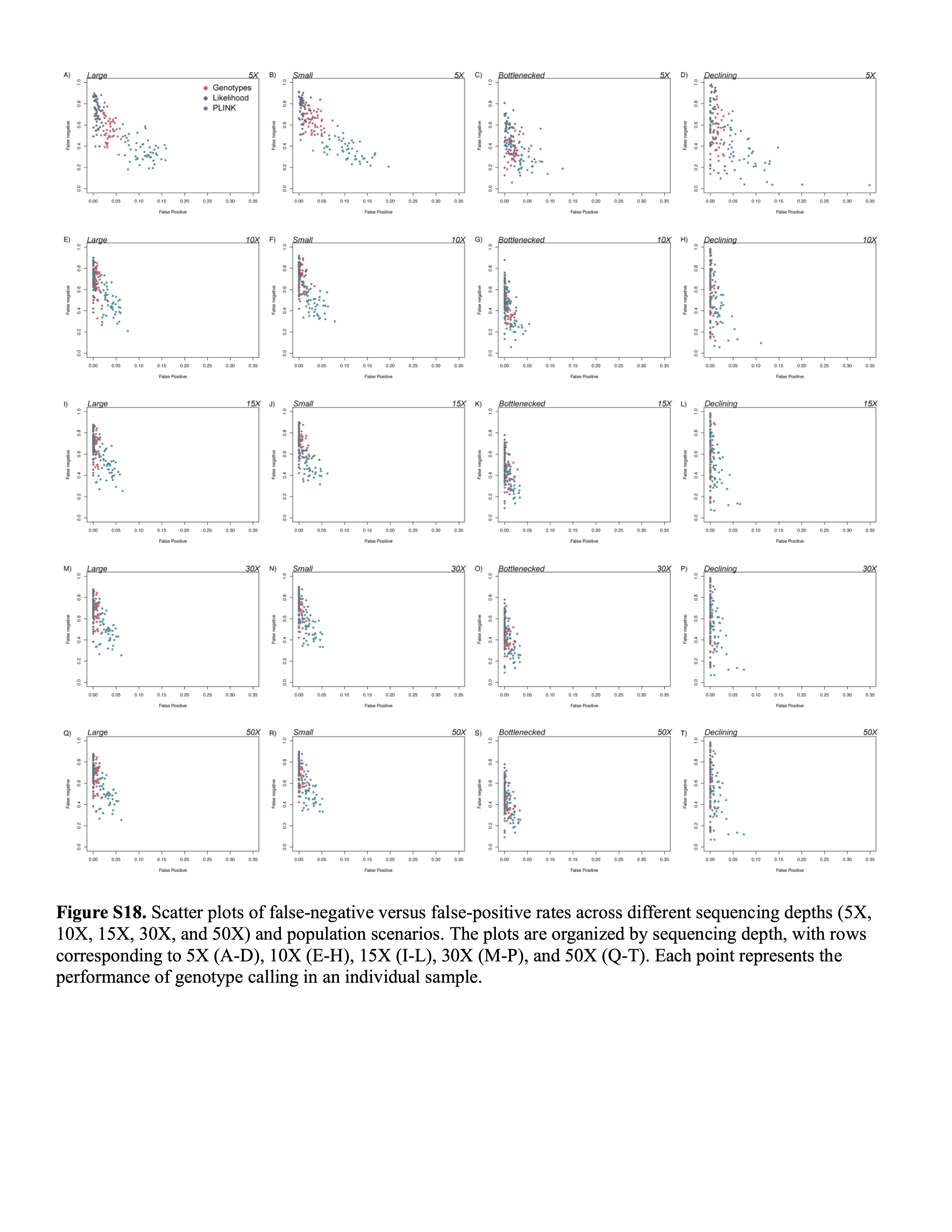

Supplement: S18 Fig — The plots are organized by sequencing depth, with rows corresponding to 5X (A-D), 10X (E-H), 15X (I-L), 30X (M-P), and 50X (Q-T). Each point represents the performance of genotype calling in an individual sample. (TIFF) [file pcbi.1012566.s024.tiff]

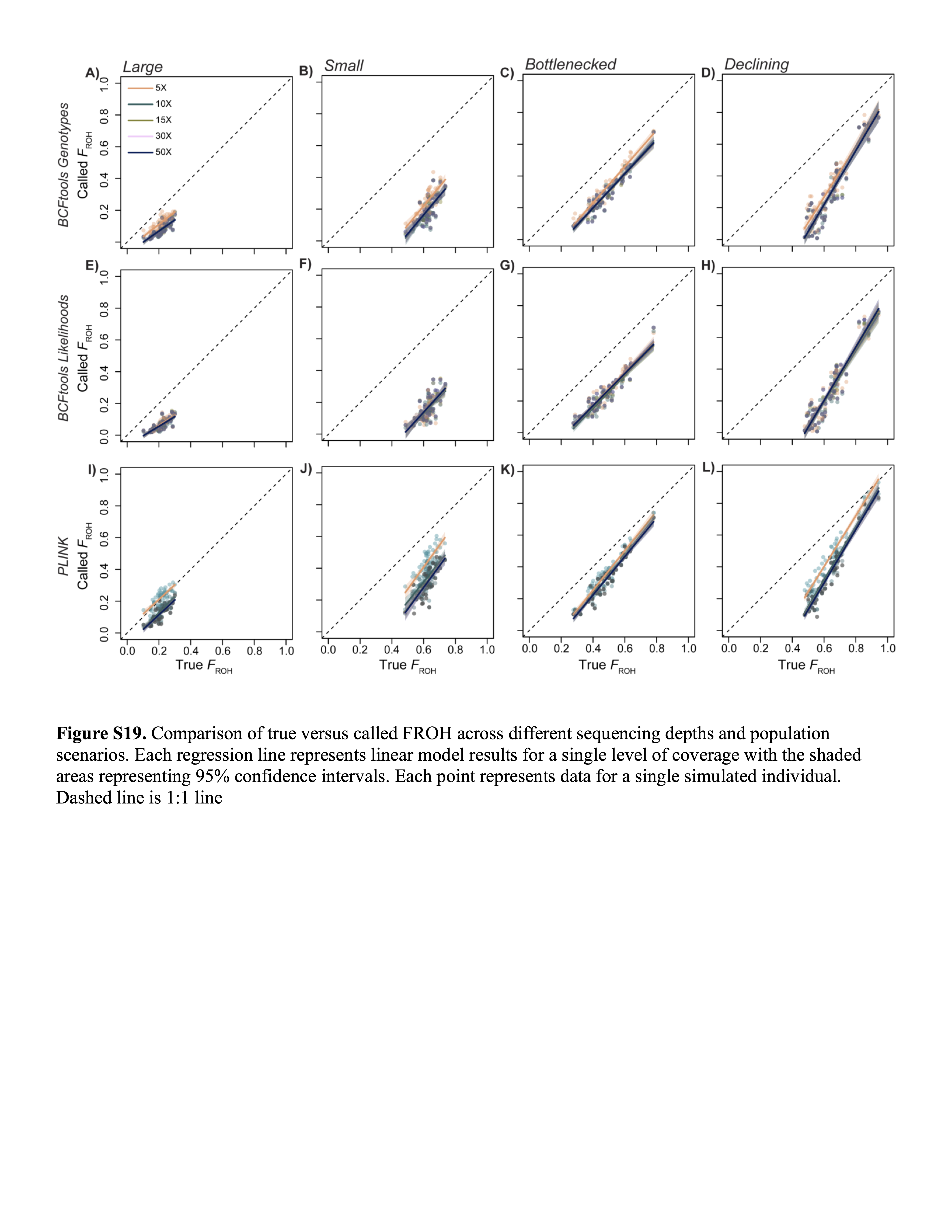

Supplement: S19 Fig — Each regression line represents linear model results for a single level of coverage with the shaded areas representing 95% confidence intervals. Each point represents data for a single simulated individual. Dashed line is 1:1 line. (TIFF) [file pcbi.1012566.s025.tiff]

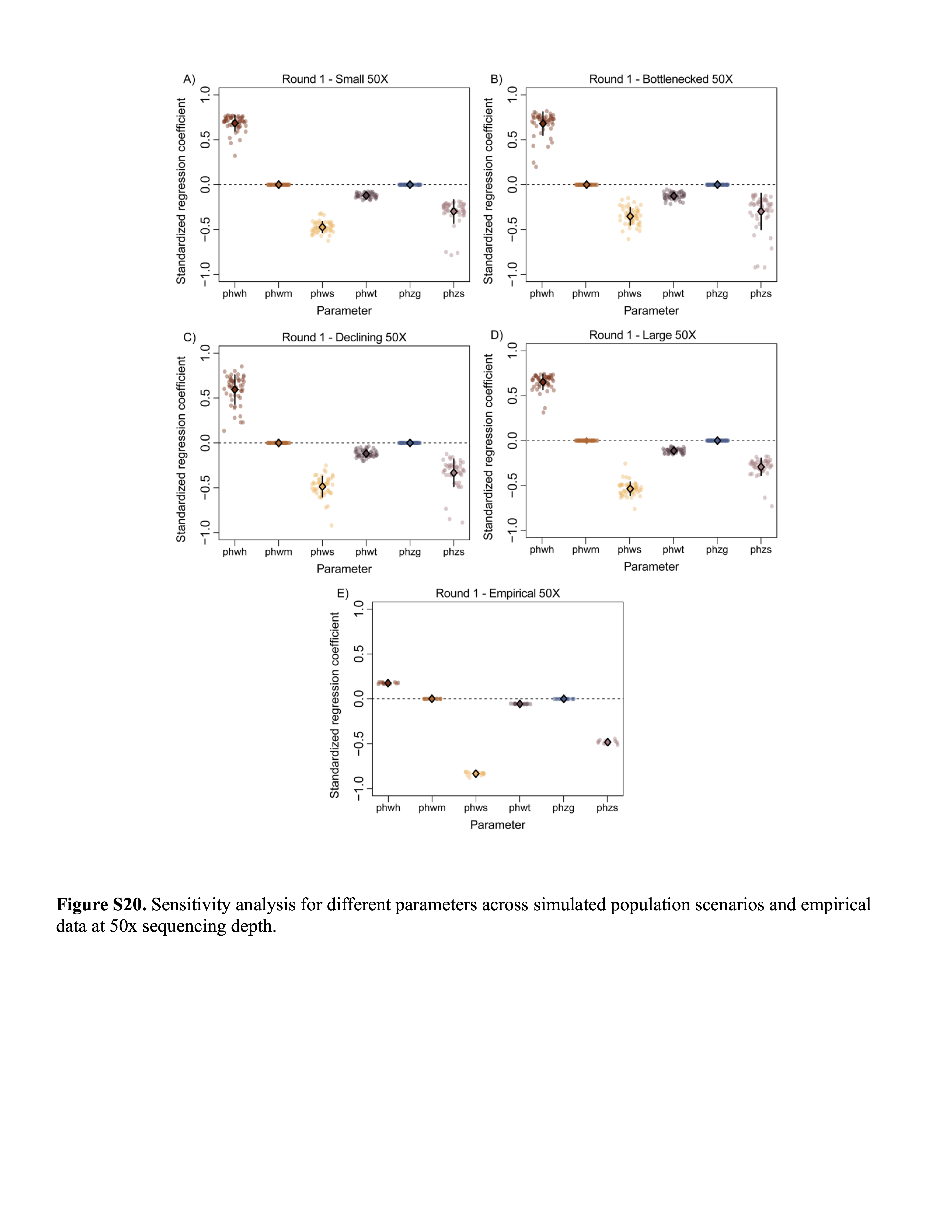

Supplement: S20 Fig — (TIFF) [file pcbi.1012566.s026.tiff]
