# Supplementary material for: Detectability of runs of homozygosity is influenced by analysis parameters and population-specific demographic history
Source: PLoS Comput Biol. 2024 Oct 31;20(10):e1012566. doi: 10.1371/journal.pcbi.1012566 (PMC11556709; doi:10.1371/journal.pcbi.1012566)
Supplement: S1 Box — (DOCX) [file pcbi.1012566.s027.docx]

**Box 1. PLINK parameter exploration through sensitivity analysis**

We applied sensitivity analysis to choose PLINK parameter values for our simulated data, here demonstrated using the declining population at 50X. In panel A, standardized regression coefficients (SRC) show the influence of each parameter on mean called FROH, with SRC > 0 indicating a positive effect (each point represents an individual). In panel B, SRC values are plotted against mean *F*_ROH_ to illustrate how the relationship changes with increasing *F*_ROH_. The tested parameter values are in the table on the right, with default settings underlined (parameter descriptions in Table 1).


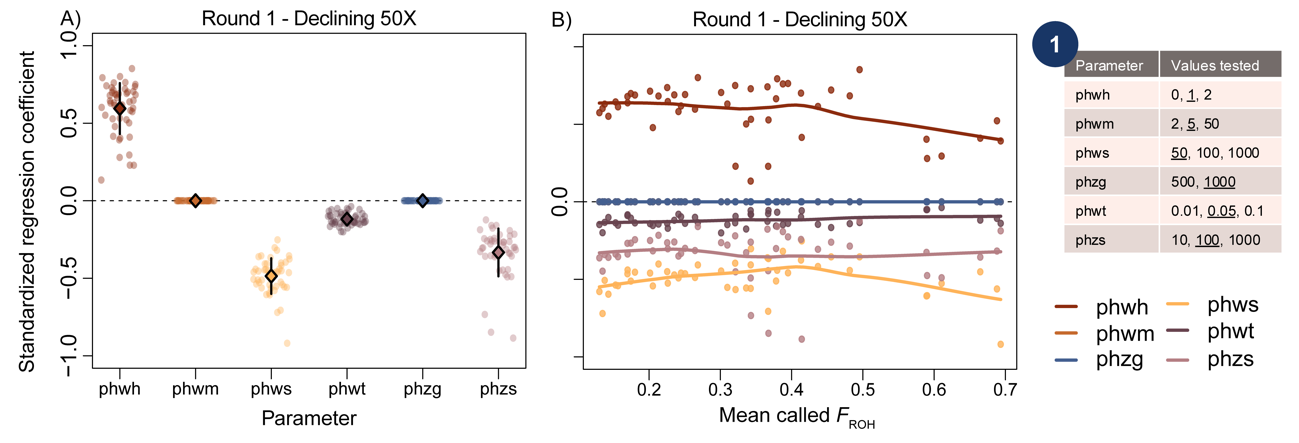


For **phwh** in Iteration 1, SRC values were positive, suggesting a positive effect on *F*_ROH_ (panel A), though the effect weakens at higher *F*_ROH_ values (panel B). To avoid inflated *F*_ROH_ values due to genotyping errors, we conservatively set **phwh** to 1. Parameters **phwm**, **phwt**, and **phzg** had little effect on *F*_ROH_, so default values were retained. The variability in **phws** and **phzs** effects (spread of points in panels A and B) suggests that further exploration of these values is needed. Testing larger values for **phws** and **phzs** (e.g., ≥ 500 for **phws** and ≥ 200 for **phzs**) resulted in no ROH calls for many individuals. Therefore, we focused on smaller ranges near the default values in Iteration 2.


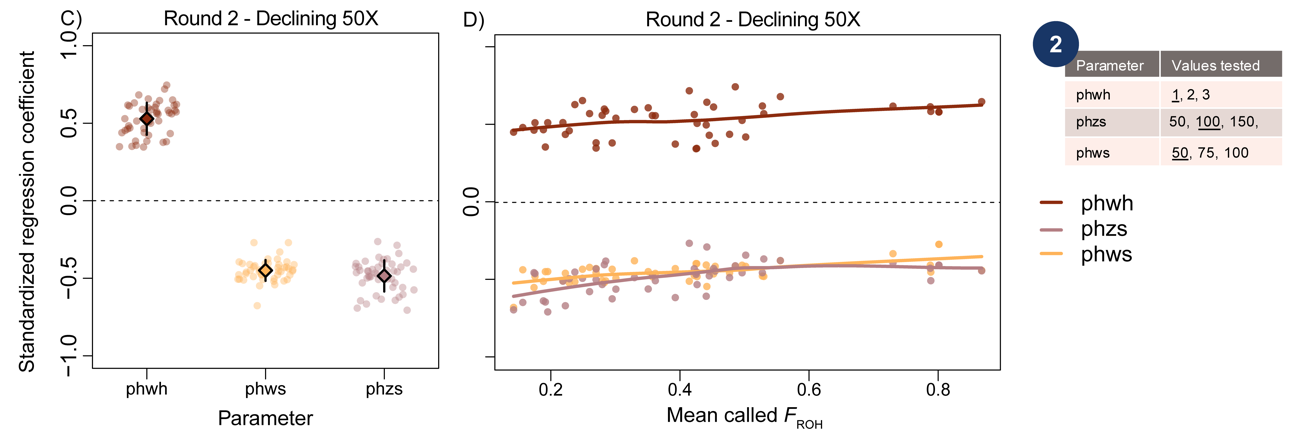


In Iteration 2, we tested higher values of **phwh**, smaller **phws** values, and less extreme values of **phzs** than in Iteration 1. Increasing **phws** still negatively impacted *F*_ROH_ but remained consistent across *F*_ROH_ values (panels C and D). Increasing **phzs** also reduced *F*_ROH_, but with less individual variation compared to Iteration 1.


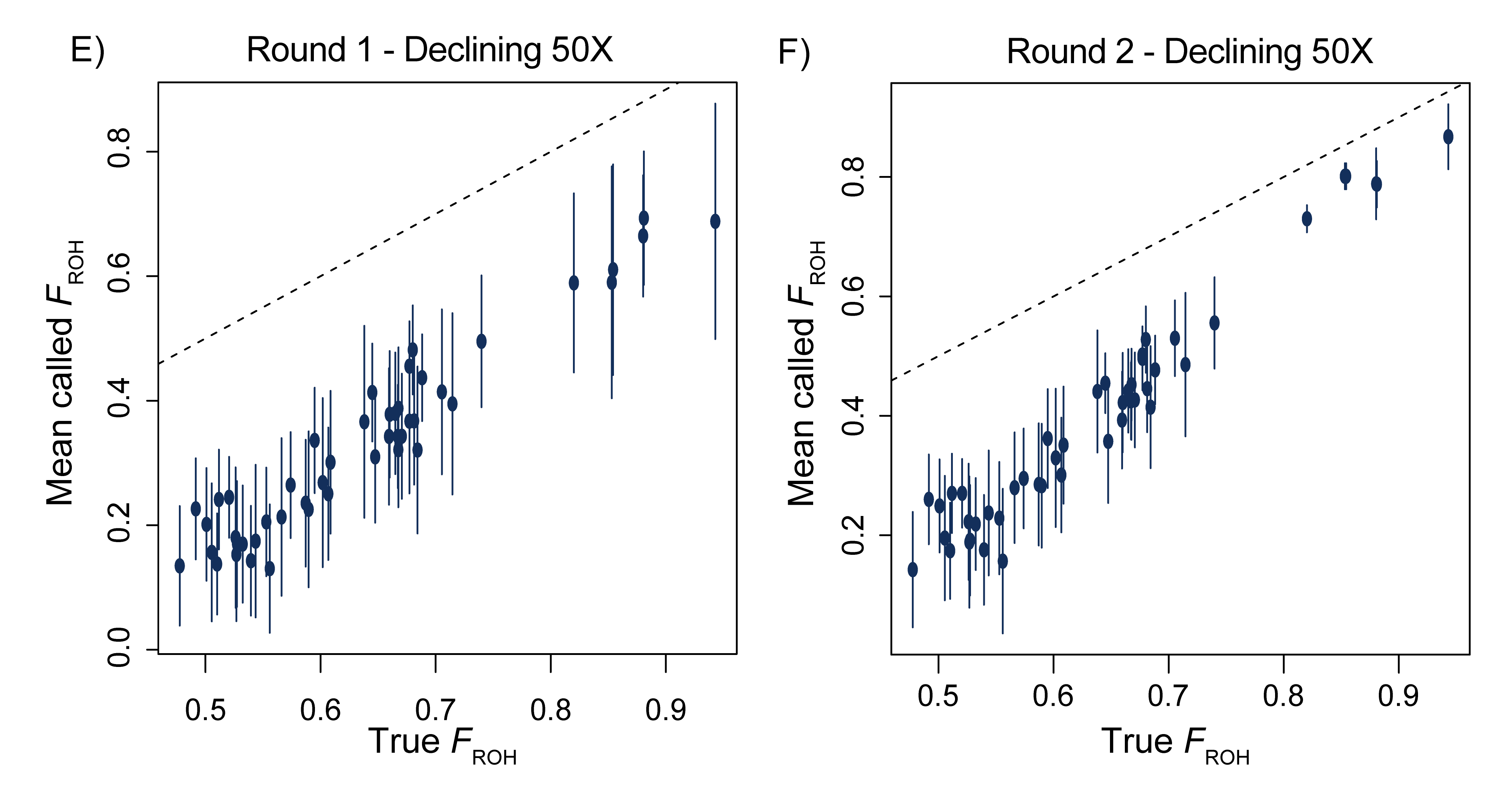


Compared to Iteration 1, Iteration 2 had smaller variation within individuals, but with no significant mean called *F*_ROH_ (panels E and F, ± SD) differences. Because the effects of **phws** and **phzs** were lower in Iteration 1 and to avoid discarding legitimate ROHs, we selected parameter values from the ranges tested in Iteration 1 (i.e., phws = 50 and phzs = 100), ultimately settling on the default values.
